# Effects of Phosphorylated-Inulin Addition on the Technological Properties of Duck-Fat-Based Whipped Cream

**DOI:** 10.3390/foods15172993

**Published:** 2026-08-26

**Authors:** Muhammad Usman Amjad, Saman Azeem, Lijuan Wang, Baocheng Xu, Yue Tian, Xinjing Dou

**Affiliations:** 1 College of Food and Bioengineering, Henan University of Science and Technology, Luoyang 471023, China; usmanamjad293@gmail.com (M.U.A.); samanazeem850@gmail.com (S.A.); pcdbb515@gmail.com (Y.T.); xinjingdou@haust.edu.cn (X.D.); 2 College of Basic Medical Science, Ningxia Medical University, Yinchuan 750004, China; lijuan.wang@nxmu.edu.cn; 3 Henan International Joint Laboratory of Food Green Processing and Safety Control, Luoyang 471023, China

**Keywords:** phosphorylated inulin, whipped cream, whipping properties, duck fat, emulsions

## Abstract

In this study, native inulin was phosphorylated and used as a stabilizer in duck-fat-based whipped cream to investigate its effects on emulsion stability, rheological properties, whipping performance, and sensory quality. Characterization of phosphorylated inulin showed a phosphorus content of 0.38% and a degree of substitution (DS) of 0.020. FTIR analysis revealed phosphate-associated absorption bands at approximately 1220 and 992 cm^−1^, attributed to P = O and P–O–C stretching vibrations, respectively, supporting the successful phosphorylation of inulin. Within the PI series, the average particle size decreased as the phosphorylated-inulin addition level increased, reaching 629 ± 5 nm in PI-12, consistent with the CLSM observations. The absolute magnitude of the negative zeta potential generally increased with the phosphorylated-inulin addition level. All emulsions exhibited shear-thinning behavior, with storage modulus (G′) exceeding loss modulus (G″). Higher phosphorylated-inulin addition levels were associated with longer whipping times and lower overrun. No measurable serum loss was detected for PI-6, PI-9, or PI-12. Air bubbles appeared smaller and more uniformly distributed at higher phosphorylated-inulin addition levels. The preliminary sensory assessment showed higher mean texture and smoothness scores for formulations containing 12 g/batch. Within the phosphorylated-inulin series, PI-6 (6 g/batch) was the lowest tested addition level at which no measurable serum loss was detected after 3 h at 22 °C, although higher addition levels further increased viscosity and viscoelastic moduli. Increasing the phosphorylated-inulin addition level above PI-6 further prolonged whipping time and reduced overrun. Further studies should evaluate storage stability and oxidative stability to validate long-term performance.

## 1. Introduction

Whipped cream is a complex three-phase emulsion system that produces a foam system with certain plasticity upon whipping. This system comprises fats, proteins, stabilizers, sweeteners, and surfactants [1,2]. Fats provide the necessary plasticity by adsorbing at the air-water interface, creating a stable, solid structure. Moreover, they contribute to the release of flavor and aroma, allowing whipped cream to meet consumers’ sensory expectations [3,4]. Many whipped cream analogues use structured vegetable fats to obtain the solid-fat characteristics required for whipping and foam stabilization [5]. However, in the present study, fractionated duck fat was selected as an alternative lipid phase to evaluate its technological suitability in a whipped cream system. Although its fatty acid composition differs from that of commonly used structured fats, nutritional quality, digestibility, lipid bioavailability, and health outcomes were not evaluated; therefore, no claim of nutritional superiority is made.

Stability issues in whipped cream include serum drainage, bubble coalescence, disproportionation, and inadequate development of the fat-mediated structural network [6]. Density differences between fat globules and the serum phase, together with interfacial membrane instability, may promote these defects, leading to poor texture and reduced shelf life [7]. To address these challenges, researchers have used various hydrocolloids as structuring agents, including xanthan gum [8], seed-derived gums [9], carrageenan-polysaccharide blends [10,11], and hydroxypropyl methylcellulose [12]. These stabilizers primarily enhance the viscosity of the continuous phase, reduce foam destabilization, and improve water retention. Although conventional hydrocolloid gums can be effective at relatively low concentrations, their effects depend on the polymer type, concentration, and interactions with the other formulation components. Excessive continuous-phase structuring may hinder air incorporation, prolong whipping time, reduce overrun, and adversely affect mouthfeel. The technological challenge is therefore to achieve sufficient foam stability without producing excessive viscosity or compromising whipping performance.

Inulin, a naturally occurring β-(2 → 1)-linked fructan polysaccharide, has attracted considerable interest due to its technological functionalities [13,14]. It has been widely used in dairy and low-fat food products as a fat replacer, texture modifier, and water-binding agent [15]. In the present study, phosphorylated inulin was evaluated solely for its technological role as a structuring and stabilizing ingredient. Prebiotic activity and other physiological properties were outside the scope of the study. The stabilizing efficiency of inulin is primarily attributed to its ability to interact with water molecules and form structured networks within the continuous phase. However, native inulin exhibits limited thickening and gel-forming ability at low concentrations [16]. Unlike highly efficient hydrocolloid gums, inulin functions mainly as a water-binding and bulk-phase structuring ingredient and may therefore require substantially higher concentrations to develop a sufficiently strong continuous-phase network. Such concentrations may increase formulation cost and adversely affect whipping performance or texture through excessive network formation.

In our previous study, native inulin was evaluated at addition levels of 0–15 g/batch in duck-fat-based whipped cream and improved its rheological properties, short-term foam stability, shaping ability, and sensory attributes [17]. The formulation containing 12 g/batch native inulin, corresponding to approximately 12.1% (*w*/*w*), provided the most suitable balance among serum-drainage resistance, rheological properties, whipping performance, and sensory quality. Based on these findings, NI-12 was selected as the native-inulin reference in the present study. However, the results of the previous study were used only to support the selection of NI-12 and not as a concurrent dose–response comparison with phosphorylated inulin.

Chemical modification of polysaccharides provides an effective approach to improve their functional properties. Phosphorylation introduces phosphate groups onto native inulin chains by replacing hydroxyl groups. This results in increased hydration capacity, enhanced polarity, and additional charged sites [14]. These structural changes may improve continuous-phase structuring, strengthen polymer-water interactions, and enhance inulin’s ability to stabilize multiphase food systems. Accordingly, the present study evaluated the effects of different phosphorylated-inulin addition levels on the physicochemical, rheological, and whipping properties of duck-fat-based whipped cream and compared phosphorylated and native inulin at the equivalent addition level of 12 g/batch.

While previous research has characterized the structural and gel properties of modified inulin [14] and its application in steamed bread [18], dough [19], and wheat starch [20], its application as a stabilizer in duck-fat-based aerated systems has not yet been fully elucidated. Therefore, the objective of this study was to investigate the effect of varying phosphorylated-inulin addition levels of 3–12 g per reference batch, corresponding to 3.34–12.15 g/100 g of the final formulation on the technological stability and quality characteristics of duck-fat-based whipped cream. Specifically, we investigated the impacts on emulsion particle size, zeta potential, rheological behavior, and whipping performance, including overrun and serum loss. Microstructure, shaping ability, and sensory attributes were also evaluated. A formulation containing native inulin and a formulation without inulin were included as experimental references, while dairy-based commercial whipped cream (CWC) was included only as an external commercial benchmark. No nutritional or physiological conclusions were drawn from the present results.

## 2. Materials and Methods

### 2.1. Materials

The fractionated duck fat used in this study was composed of 45.33% saturated fatty acids, 33.23% monounsaturated fatty acids, and 21.44% polyunsaturated fatty acids, and was kindly supplied by Henan Huaying Cherry Valley Co., Ltd. (Xinyang, China). Natural inulin derived from chicory root, with a degree of polymerization between 2 and 60 and a purity >86%, was purchased from Cosucra (Warcoing, Belgium). Mono- and diglycerides were obtained from Jialishi Additives Co., Ltd. (Hai’an, China). Sodium caseinate (≥90% protein content) was supplied by Huaan Group (Linxia, China). Polyglycerol fatty acid ester (HLB 13) was provided by Zhengzhou Dahe Food Technology Co., Ltd. (Zhengzhou, China). Disodium hydrogen phosphate, sodium dihydrogen phosphate, potassium dihydrogen phosphate, urea, sulfuric acid, nitric acid, ammonium molybdate, and sodium sulfite were acquired from Tianjin De’en Chemical Reagent Co., Ltd. (Tianjin, China). Analytical-grade reagents were used throughout the experiments. The fluorescent dyes Nile red and Nile blue were purchased from Shanghai Yuanleaf Biological Technology Co., Ltd. (Shanghai, China). A commercial dairy fat-based whipping cream was sourced from Fonterra Commercial Trading Co., Ltd. (Shanghai, China). Deionized water was prepared using a Milli-Q purification system (Millipore Corporation, Milford, MA, USA).

### 2.2. Preparation of Phosphorylated Inulin

Phosphorylated inulin was prepared using a modified dry-heating approach based on the method of Chen et al. [14], with slight adjustments. Initially, a phosphorylation solution was prepared by dissolving 0.5 g of disodium hydrogen phosphate, 0.5 g of sodium dihydrogen phosphate, and 0.4 g of urea in 20 mL of distilled water. The pH of the solution was adjusted to 6.5 using either 1 mol/L NaOH or HCl as required. Subsequently, 10 g of native inulin was gradually added to the prepared solution and stirred continuously for 30 min at 35 °C to ensure uniform dispersion. The mixture was then transferred to an electric hot-air drying oven and subjected to thermal treatment at 120 °C for 4 h to induce phosphorylation. After completion of the reaction, the solution was cooled to room temperature. The crude product was subjected to three successive washing and filtration cycles using 95% ethanol as a purification step intended to reduce unreacted reagents and low-molecular-weight by-products. The recovered solid was then dried at 40 °C for 12 h, finely ground, and passed through a 100-mesh sieve to obtain phosphorylated inulin powder.

### 2.3. Determination of Phosphorus Content and Degree of Substitution

The phosphorus content of phosphorylated inulin (PI) was quantified using the molybdenum blue spectrophotometric method according to Wu et al. [21] with slight modifications. A 0.5 g portion of PI was combined with 7.5 mL of a sulfuric acid–nitric acid mixture (1:1, *v*/*v*) and heated until digestion was complete and a clear solution was obtained. After cooling to room temperature, the digested solution was mixed with 2 mL of ammonium molybdate (50 g/L), 1 mL of sodium sulfite (200 g/L), and 1 mL of hydroquinone (5 g/L). The mixture was brought to a final volume of 25 mL with distilled water and maintained at room temperature for 30 min. Absorbance was subsequently recorded at 660 nm. Phosphorus concentration was determined from a calibration curve prepared with potassium dihydrogen phosphate over a concentration range of 0–8.0 μg/mL. The degree of substitution (DS) was calculated as follows:

(1) $${DS}_{P}=\;\frac{162\;\left(P\right)}{3100-103(P)}$$

where (P) denotes the phosphorus content (%) determined colorimetrically, 162 is the molar mass of an anhydrofructose unit, 3100 represents the atomic mass of phosphorus multiplied by 100, and 103 is the molar mass of the introduced phosphate group.

### 2.4. Fourier-Transform Infrared (FTIR) Spectroscopy Analysis

The structural features of native inulin (NI) and phosphorylated inulin (PI) were characterized by Fourier-transform infrared (FTIR) spectroscopy (Bruker Corporation, Berlin, Germany). Before sample preparation, potassium bromide (KBr) was oven-dried at 105 °C for 24 h to remove residual moisture. Each powdered sample was mixed thoroughly with the dried KBr at a sample-to-KBr mass ratio of 1:100 and finely ground under a heating lamp to obtain a homogeneous powder. The resulting mixture was compressed at 15 MPa for 30 s using a tablet press to produce a translucent pellet, which was analyzed immediately. The spectra were collected over the wavenumber range of 4000–400 cm^−1^ at a resolution of 4 cm^−1^, with 64 scans accumulated for each spectrum.

### 2.5. Preparation of Whipping Cream Emulsions and Whipped Cream

The pH of deionized water was adjusted to 7.0 using potassium dihydrogen phosphate and disodium hydrogen phosphate. The base formulation contained 56.17 g deionized water, 29.50 g fractionated duck fat, 0.50 g sodium caseinate, 0.10 g polyglycerol fatty acid ester, and 0.50 g mono- and diglycerides. These quantities were held constant in all laboratory-prepared formulations. Phosphorylated inulin was added at 3, 6, 9, or 12 g per batch to prepare PI-3, PI-6, PI-9, and PI-12, respectively. NI-12 contained 12 g native inulin, whereas the negative control (NC) contained no inulin. The NC was not balanced by adding additional water; therefore, the total batch masses were 86.77, 89.77, 92.77, 95.77, and 98.77 g for NC, PI-3, PI-6, PI-9, and PI-12, respectively, and 98.77 g for NI-12. The actual quantities and corresponding compositions normalized to 100 g are presented in Table 1A,B. For experimental preparation, each formulation was proportionally scaled to obtain 200 g of emulsion for subsequent analyses without altering the ingredient ratios. Because inulin was added without replacing water, the total solids increased and the relative water proportion decreased across the formulations. The treatment codes therefore represent the amount of inulin added per batch rather than its final percentage by mass.

The aqueous phase was prepared by dispersing sodium caseinate, polyglycerol fatty acid ester, and the specified amount of inulin in the pH-adjusted water. The mixture was heated to 60 °C and stirred continuously until homogeneous. Fractionated duck fat was separately heated to 70 °C and maintained for 30 min to ensure complete melting, after which mono- and diglycerides were added and dissolved under continuous mixing. The oil and water phases were pre-emulsified at 10,000 rpm for 3 min using an FJ200-SH digital high-speed shear mixer (Shanghai Specimen Model Factory, Shanghai, China) fitted with a cylindrical, slotted rotor–stator working head. The resulting coarse emulsion was subjected to one cycle of high-pressure homogenization at 15 MPa for 3 min using a GJJ-0.02/60 homogenizer (Shanghai Noni Light Industry Machinery Co., Ltd., Shanghai, China). The temperature during and immediately after homogenization was not recorded. The emulsions were subsequently stored at 4 °C for 24 h before rheological analysis and whipping.

After storage at 4 °C for 24 h, 150 g of each emulsion was transferred to a pre-cooled stainless-steel bowl with an internal diameter of 20 cm, a height of 17 cm, and an approximate capacity of 3.3 L. The samples were whipped at speed setting 10 using a planetary stand mixer (model 5KSM3311; KitchenAid, St. Joseph, MI, USA) fitted with a six-wire whisk attachment. The temperature of each emulsion immediately before whipping was approximately 4 °C. During whipping, ice packs were placed around the bowl to limit the increase in sample temperature. The whipping endpoint was defined as the point at which the whipped cream formed a firm peak that retained its shape without collapse or flow for at least 30 s after the whisk was lifted vertically from the sample. The elapsed time from the start of whipping to the endpoint was recorded as the whipping time. All samples were whipped by the same trained operator to minimize variability associated with visual identification of the endpoint. Three independently prepared batches of each formulation were whipped under identical operating conditions, and whipping time was reported as the mean ± standard deviation.

### 2.6. Average Particle Size and Zeta Potential of Emulsions

The mean particle size and zeta potential of the emulsions were measured using a BeNano 90 Zeta particle-size and zeta-potential analyzer (Bettersize Instruments Ltd., Dandong, China). Prior to analysis, each sample was diluted 1000-fold with deionized water to reduce multiple scattering effects. After equilibration at 25 ° C for 5 min, measurements were performed at a scattering angle of 90°. The refractive index was set to 1.33 for the continuous phase (water) and 1.451 for the dispersed phase, with an absorption index of 0.001 [22]. Each sample was analyzed in triplicate, and the results are reported as mean values.

### 2.7. Confocal Laser Scanning Microscopy (CLSM)

Confocal laser scanning microscopy (CLSM) was performed using an Olympus Fluoview FV3000 microscope (Olympus, Tokyo, Japan) to examine the microstructure of the emulsions and visualize the distribution of lipid droplets and protein-rich regions. Nile Red (0.1%, *w*/*w*) was dissolved in acetone to stain the lipid phase, whereas Nile Blue (0.5%, *w*/*w*) was dissolved in absolute ethanol to stain the protein-rich regions. Images were acquired using a 63 × oil-immersion objective with 3 × digital zoom. Nile Red and Nile Blue were excited at 552 and 633 nm, respectively, with fluorescence emissions collected over 570–620 and 650–749 nm, respectively.

### 2.8. Rheological Measurements

Rheological behavior of the emulsions was analyzed using a TA DHR-2 rheometer (TA Instruments, Milford, MA, USA) equipped with a 40 mm parallel steel plate and a 1000 μm geometry gap. Before measurements, all samples were equilibrated at 4 °C for 24 h. Approximately 2 mL of each sample was carefully placed onto the peltier plate maintained at 4 °C. Steady-state shear viscosity was measured over a logarithmic shear rate range of 0.1 to 100 s^−1^, with data points recorded every 5 s following a 30 s equilibration period. The steady-shear data from each independent batch were fitted to the power-law model using equation:

(2) $$\tau =K{\dot{\gamma }}^{n}$$

where τ is shear stress (Pa), $\dot{\gamma }$ is shear rate (s^−1^), K is the consistency index (Pa·s^n^), and n is the flow behavior index. The model parameters were determined by linear regression of ln(τ) against ln($\dot{\gamma }$), and goodness of fit was evaluated using R^2^.

In addition, an oscillatory strain sweep ranging from 0.01% to 100% at a constant frequency of 1.0 Hz was conducted to determine the linear viscoelastic region (LVR) and to evaluate storage modulus (G′) and loss modulus (G″) [23]. The LVR was defined as the strain range over which (G′) remained approximately independent of strain, and its upper limit was taken as the strain at which (G′) decreased by 5% from its low-strain plateau value [24,25]. Frequency sweeps were subsequently performed within the LVR at a constant strain of 0.05% over an angular-frequency range of 0.1–10 rad s^−1^. The resulting (G′) and (G″) values were recorded, and the presence of a modulus crossover was assessed from their intersection.

### 2.9. Whipping Time

Whipping time was recorded, in seconds, from the initiation of mixing at speed setting 10 until the predefined whipping endpoint described in Section 2.5 was reached [3].

### 2.10. Overrun

The overrun of whipped samples was determined using a slightly modified method based on that reported by Zeng et al. [26]. The same pre-weighed aluminum container was filled to the same level with either unwhipped emulsion or whipped cream collected at the predefined whipping endpoint. The sample surface was levelled before weighing, without compressing the whipped cream. Overrun was calculated as follows:

(3) $$Overrun\;\left(\%\right)=\;\frac{M_{1}-M_{2}}{M_{2}}\;\times 100$$

where *M*_1_ and *M*_2_ are the net masses (g) of equal volumes of unwhipped emulsion and whipped cream, respectively. Measurements were performed for three independently prepared batches of each formulation (*n* = 3).

### 2.11. Determination of Serum Loss

To determine serum loss, a 20 g portion of the optimally whipped cream was placed on a 20-mesh sieve and incubated at 22 °C for 3 h in a temperature-controlled chamber. The percentage serum loss was calculated using Equation (4):

(4) $$Serum\;loss\;\left(\%\right)=\;\frac{m_{serum}}{\;m_{whipped}}\times 100$$

where m_serum_ denotes the mass of the serum separated from the whipped cream after 3 h of incubation, and m_whipped cream_ represents the initial mass of the whipped cream.

### 2.12. Optical Microscopy of Whipped Cream

The microstructure of whipped cream was observed using an optical microscope (Changfang Optical Instruments Co., Ltd., Shanghai, China). The whipped cream samples obtained at the optimal whipping time were carefully placed on glass slides, covered with a coverslip, and visualized at 40 × magnification. The microscopic images were analyzed to evaluate the size and distribution of air bubbles within the foam structure.

### 2.13. Shaping Ability

A total of 50 g of freshly whipped cream was transferred with a rubber spatula into a disposable pastry bag fitted with a nozzle. The sample was then extruded through a nozzle to facilitate molding. Gentle manual pressure was applied to shape the whipped cream, and its ability to maintain the molded shape was carefully observed. Finally, the molded samples were photographed to document their appearance and textural characteristics.

### 2.14. Cross-Sectional View

The structural stability of the whipped cream was evaluated by analyzing the surface characteristics of its cross-sectional structure. Freshly prepared whipped samples were shaped into a mound and equilibrated at 25 °C for 1 h. Following equilibration, the samples were carefully cut using a metal scraper to expose the internal structure. The resulting cross-section was immediately photographed under consistent lighting conditions.

### 2.15. Sensory Evaluation

A laboratory-based sensory acceptance evaluation was conducted with 20 volunteer assessors aged 22–45 years who regularly consumed whipped cream or related products. Volunteers were recruited based on the following inclusion criteria: normal sensory acuity, no known allergies to or intolerances of dairy- or fat-based products, and regular consumption of whipped cream or similar confectionery products. Individuals who smoked or had respiratory illnesses or other conditions affecting taste or odor perception were excluded. Before the evaluation, the assessors were familiarized with the evaluated attributes and scoring procedure but did not undergo formal descriptive-panel training. The panel size was selected for preliminary laboratory screening and was not intended to provide population-level estimates of consumer acceptance.

All seven formulations were evaluated in each of three separate sensory sessions using independently prepared sample batches. In each session, every assessor received 10 g of each formulation served at 8 ± 1 °C. Samples were identified using random three-digit codes and presented in a randomized order. Evaluations were conducted in individual sensory booths under consistent lighting at an ambient temperature of 22 ± 1 °C. Clean drinking water was provided, and assessors were instructed to rinse their mouths between consecutive samples.

Texture, overall flavor quality, sweetness, and smoothness were evaluated using a 5-point acceptance scale ranging from 1 (poor) to 5 (excellent). Overall flavor quality represented an integrated assessment of taste and aroma rather than the intensity of a particular flavor or off-flavor. All assessors provided informed consent before participation. For each attribute and formulation, the individual assessor ratings were averaged within each session. The results are presented as the mean ± standard deviation of the three session-level mean scores. Although sample presentation was randomized, individual assessor identifiers and exact presentation sequences were not retained.

### 2.16. Statistical Analysis

Each formulation was independently prepared on three separate occasions, resulting in three independent experimental batches (*n* = 3). All analyses were conducted separately for each batch, and the results are expressed as mean ± standard deviation. Statistical differences among samples were evaluated using one-way analysis of variance (ANOVA), followed by Tukey’s multiple comparison test at a significance level of *p* < 0.05. Serum-loss results containing non-detectable values were reported descriptively. Statistical analysis was performed using IBM SPSS Statistics 27, while graphs and data visualization were generated using Origin 2025.

## 3. Results and Discussion

### 3.1. Degree of Substitution and FTIR Characterization

The phosphorus content of phosphorylated inulin was 0.38%. Based on this value, the degree of substitution (DS) was calculated to be 0.020, indicating that a relatively small proportion of the hydroxyl groups in the inulin molecules was substituted with phosphate groups. However, the relatively low DS suggests that phosphorylation occurred to a limited extent.

**Figure 1:**
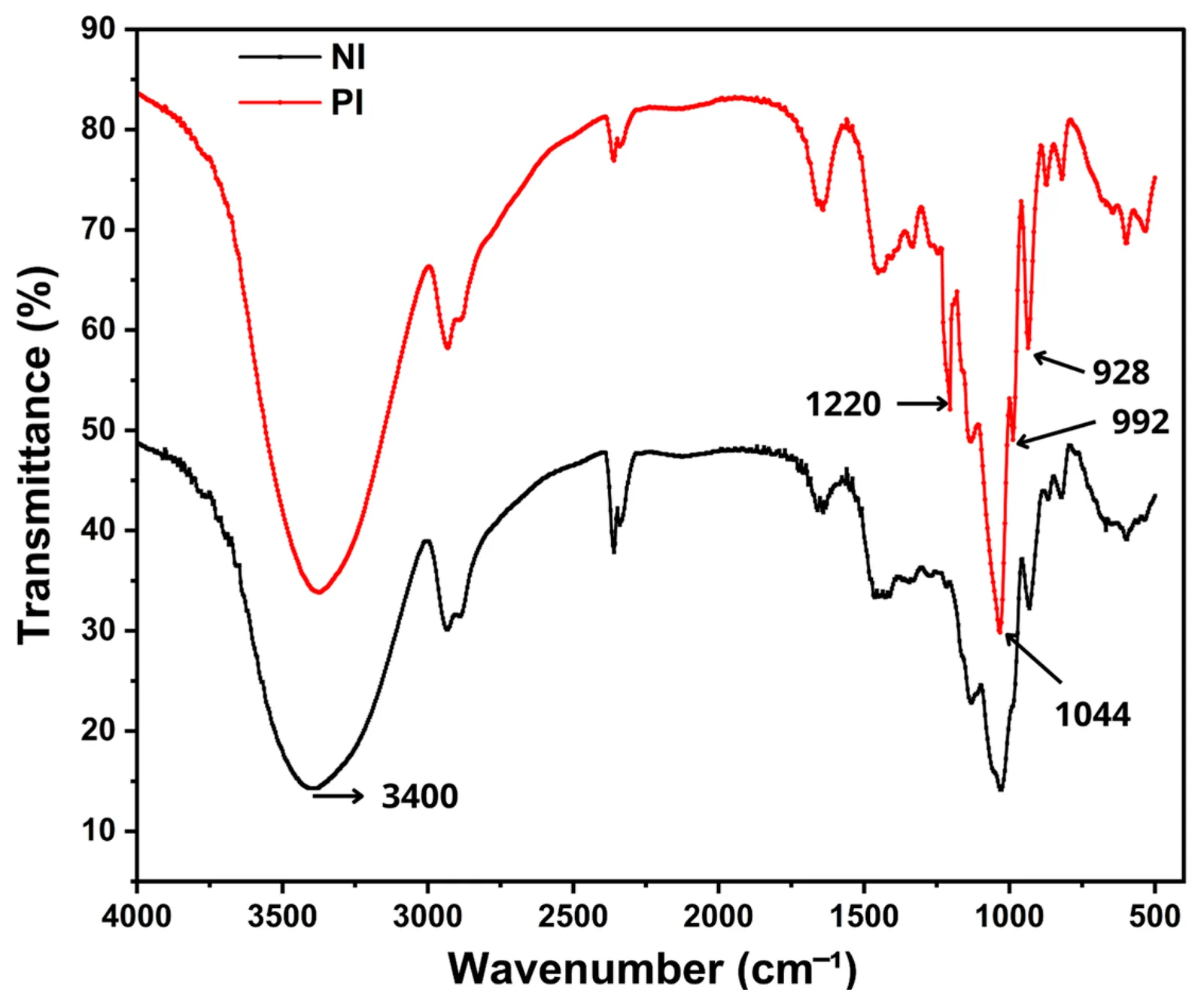
Fourier-transform infrared (FTIR) spectra of native inulin (NI) and phosphorylated inulin (PI).

The FTIR spectra of native inulin (NI) and phosphorylated inulin (PI) are presented in Figure 1. Both samples exhibited a broad absorption band at approximately 3400 cm^−1^, corresponding to O–H stretching vibrations. The characteristic peaks observed at approximately 1044 cm^−1^ and 928 cm^−1^ were attributed to C–O/C–O–C stretching vibrations of the fructan skeleton and the β-(2 → 1) glycosidic linkage of inulin, respectively [27]. The retention of these characteristic bands in PI indicates that the phosphorylation treatment did not substantially disrupt the fundamental structure of inulin. Compared with NI, PI exhibited distinct absorption features at approximately 1220 and 992 cm^−1^, which were attributed to P = O and P–O–C stretching vibrations, respectively. These phosphate-associated bands support the successful phosphorylation of inulin, suggesting that some hydroxyl groups were substituted with phosphate groups [28,29]. This finding is consistent with the measured phosphorus content of PI (0.38%) and its corresponding degree of substitution (DS = 0.020). Collectively, the phosphate-associated FTIR features, phosphorus content, and calculated DS support the successful introduction of a relatively small number of phosphate groups into the inulin molecules.

Although repeated ethanol washing, filtration, and drying were carried out to purify phosphorylated inulin, residual free phosphate and urea were not analytically quantified in the final powder. Consequently, their complete removal cannot be confirmed, and the measured total phosphorus content and FTIR spectra should not be interpreted as evidence of product purity. The whipped cream samples containing phosphorylated inulin were included in the exploratory sensory evaluation because the phosphorylated inulin was prepared according to the published procedure [14] and subjected to repeated purification steps; however, this process-based assessment did not substitute for analytical verification of residual reagents. Residual reagent analysis is therefore required before further food applications.

### 3.2. Average Particle Size and Zeta Potential

**Figure 2:**
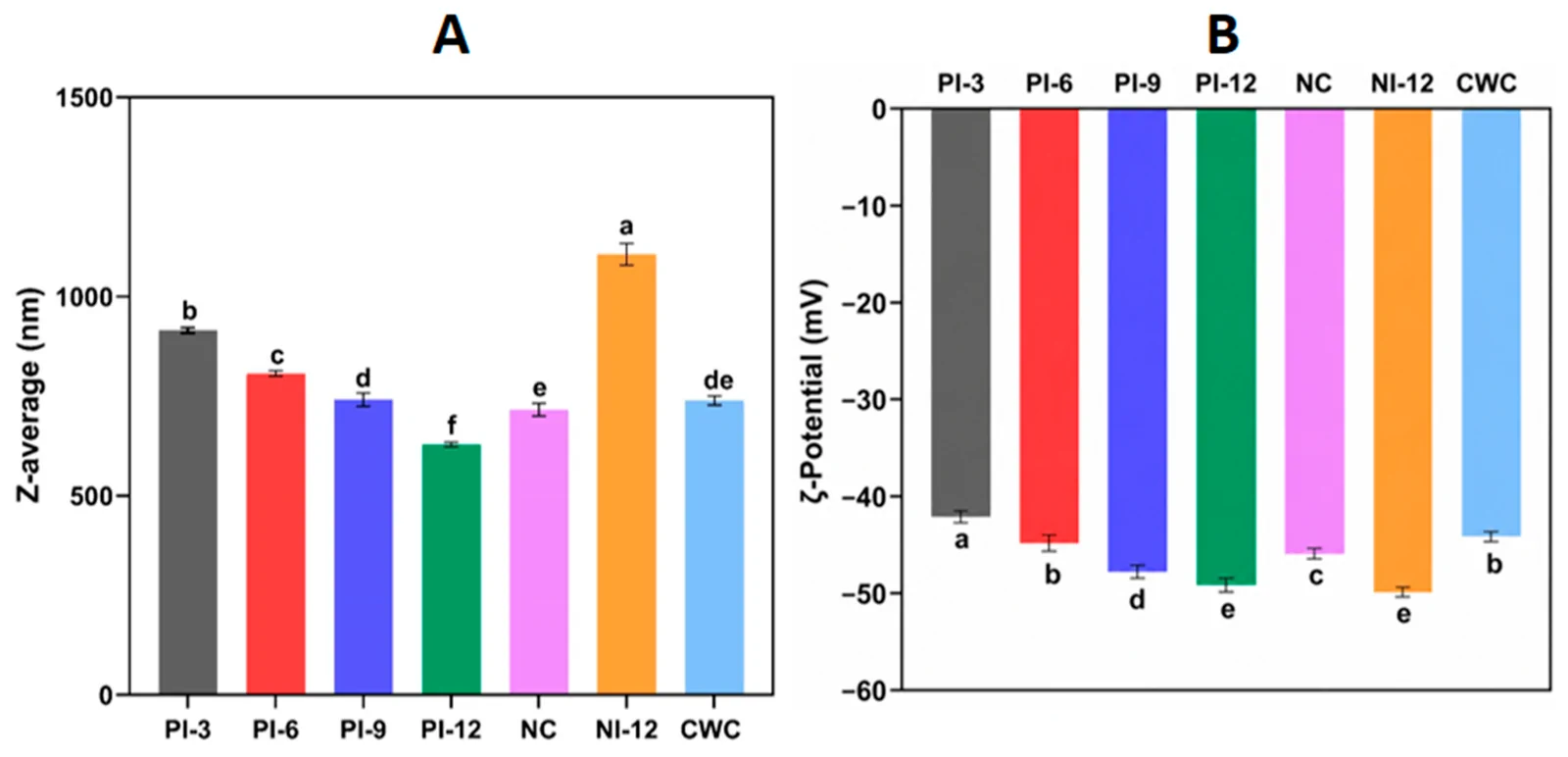
(**A**) Z-average particle size and (**B**) Zeta potential of the whipping-cream emulsions. Samples included the duck-fat-based formulation without inulin (NC), formulations containing phosphorylated inulin (PI-3–PI-12), the formulation containing native inulin (NI-12), and commercial whipping cream (CWC). Different superscript letters (a–f) indicate significant differences among samples (*p* < 0.05).

The average particle size of emulsion droplets is an important structural characteristic that may influence physical stability after preparation, with smaller average particle sizes potentially contributing to greater stability [30,31]. The average particle sizes of samples measured after 1000-fold dilution are presented in Figure 2A. Among the samples stabilized with phosphorylated inulin, average particle size decreased from 915.66 ± 7.02 nm in PI-3 to 629 ± 5 nm in PI-12. Although particle size decreased as the phosphorylated-inulin addition level increased, samples with lower addition levels did not consistently exhibit smaller particles than NC. The reduction in average particle size observed at higher phosphorylated inulin addition levels may partly be associated with the increased viscosity of the continuous phase, which could restrict droplet mobility and reduce collision and coalescence. Enhanced hydration associated with phosphate groups may also have contributed to emulsion stabilization, although this mechanism was not directly evaluated in the present study. Among all samples, NI-12 exhibited the largest average particle size (1106 ± 27.87 nm), exceeding that observed for PI-12. This suggests that phosphorylated inulin produced a smaller average particle size than native inulin under the present experimental conditions. The smaller particles may facilitate interactions among phosphorylated inulin, proteins, and water molecules, which could contribute to improved emulsion stability [14]. It should be noted that all emulsions were diluted 1000-fold with deionized water to minimize multiple-scattering effects. However, this dilution may have altered particle interactions and aggregation. Therefore, the measured particle sizes should be interpreted as comparative values obtained under diluted conditions rather than as a direct representation of the original emulsions.

The magnitude of the zeta potential provides information about electrostatic interactions among emulsion droplets. A greater absolute magnitude may indicate stronger electrostatic repulsion [32,33]. However, zeta potential alone does not establish overall emulsion stability. The zeta potential values of the emulsions are shown in Figure 2B. The absolute magnitude of the negative zeta potential generally increased with the phosphorylated-inulin addition level. PI-12 and NI-12 exhibited comparable magnitudes of negative zeta potential. However, these values may also have been influenced by pH, ionic strength, sodium caseinate, emulsifiers, buffer salts, possible residual phosphate species, and the 1000-fold dilution. Because the interfacial adsorption of phosphorylated inulin was not directly measured, the observed trend should not be attributed solely to the adsorption of phosphorylated inulin at the droplet interface.

### 3.3. Microstructure of Emulsions by CLSM

**Figure 3:**
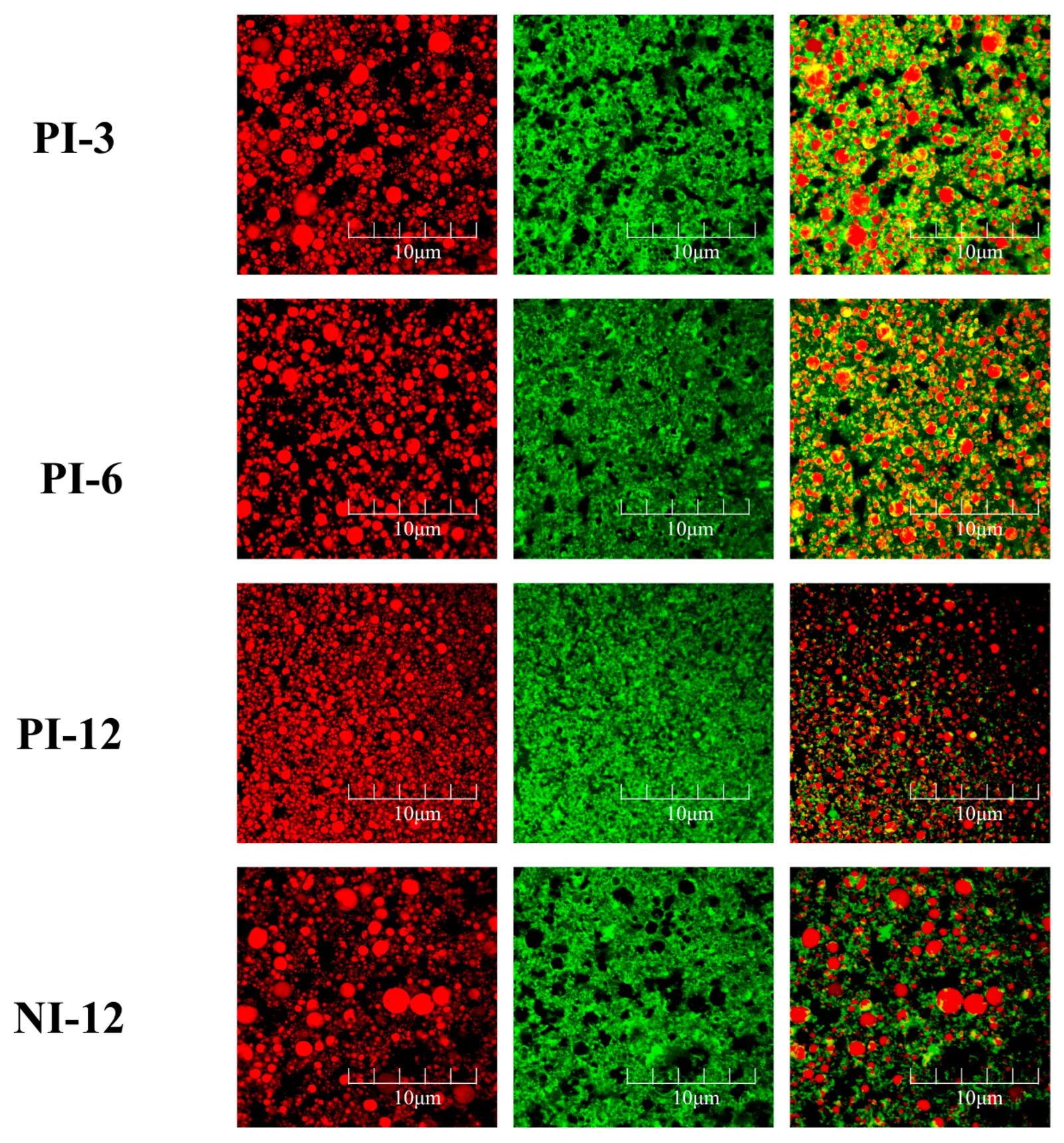
Confocal laser scanning microscopy images of duck-fat-based whipping-cream emulsions containing phosphorylated inulin (PI-3, PI-6, and PI-12) and native inulin (NI-12). Fat droplets were stained with Nile Red (red), and the protein phase was stained with Nile Blue (green). Scale bar = 10 μm.

The distribution of fat droplets (red) and proteins (green) in emulsions under CLSM is shown in Figure 3. Proteins (green) appeared to be distributed around the fat droplets (red), which may contribute to limiting droplet coalescence. Some fat droplets appeared non-spherical in the displayed CLSM fields; however, droplet shape was evaluated only qualitatively. Fat droplets generally appeared smaller as the phosphorylated-inulin addition level increased, with PI-12 showing smaller apparent droplets than PI-3, PI-6, and NI-12. This apparent variation in droplet size across the presented CLSM images may be due to increased emulsion viscosity. In addition, the introduction of phosphate groups may increase the water affinity of phosphorylated inulin and contribute to continuous-phase structuring, although this mechanism was not directly evaluated in the present study. Droplets in PI-12 also appeared more densely arranged than those in PI-3, PI-6, and the native-inulin reference, NI-12. In conclusion, these qualitative observations suggest that phosphorylated inulin influenced the emulsion microstructure as a continuous-phase structuring ingredient rather than functioning as an emulsifier.

### 3.4. Rheological Properties

#### 3.4.1. Viscosity

**Figure 4:**
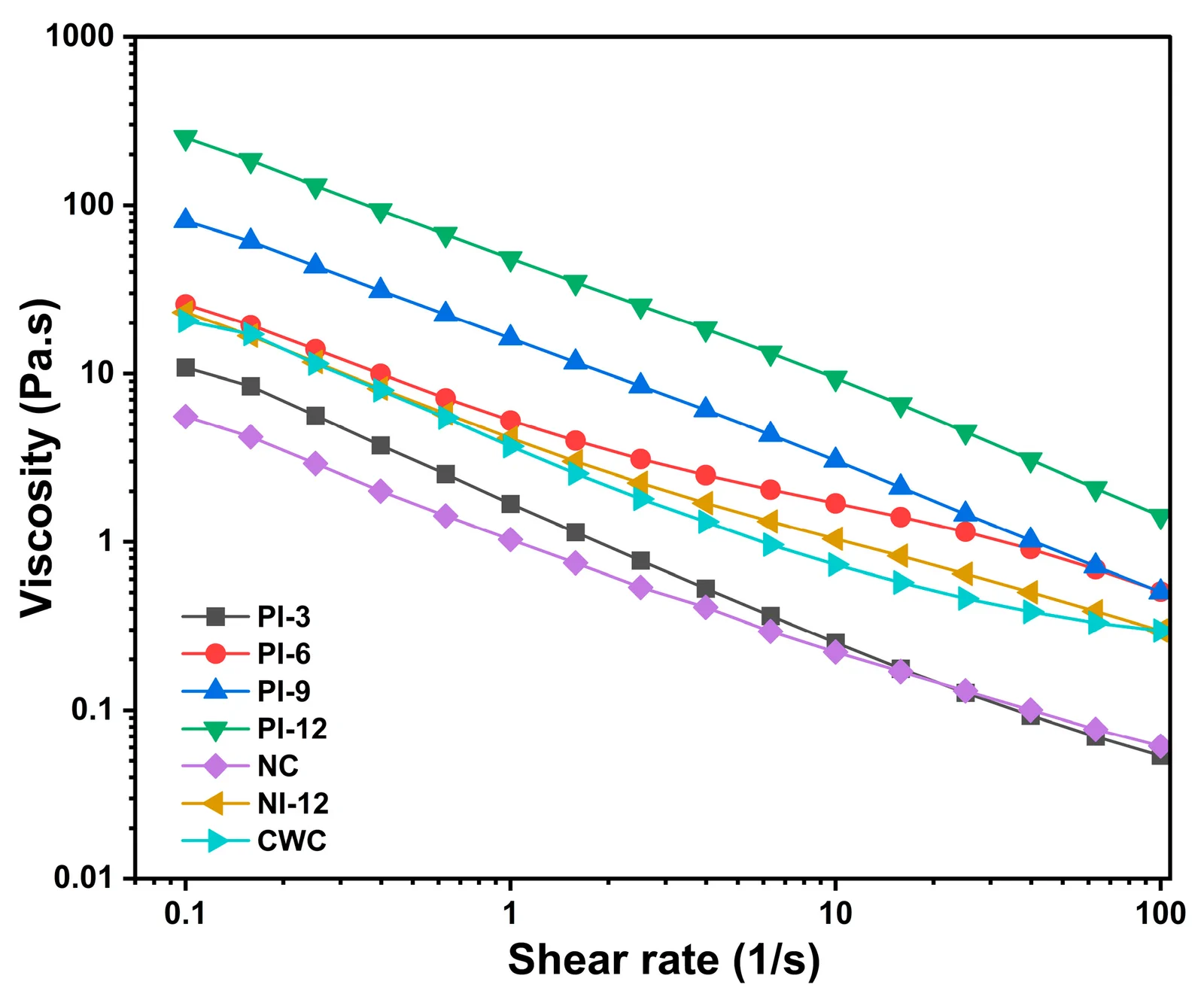
Viscosity as a function of shear rate for duck-fat-based whipping-cream emulsions formulated without inulin (NC), with phosphorylated inulin (PI-3–PI-12), and with native inulin (NI-12), together with commercial whipping cream (CWC). Measurements were conducted at 4 °C over a shear-rate range of 0.1–100 s^−1^.

Viscosity is a critical determinant of the quality of whipped cream. Increased viscosity effectively retards particle sedimentation and inhibits creaming phenomena, thereby significantly enhancing the overall storage stability of the emulsion [34,35]. However, higher viscosity may impede air incorporation and reduce the overrun of the whipped cream. In contrast, excessively low viscosity can facilitate air incorporation during whipping, leading to a higher overrun. However, it may also promote the coalescence of adjacent air bubbles and increase bubble pressure, ultimately leading to foam disproportionation [36]. As shown in Figure 4, all samples exhibited shear-thinning behavior over the shear-rate range of 0.1–100 s^−1^, with apparent viscosity decreasing as the shear rate increased. This pseudoplastic response may reflect shear-induced disruption or rearrangement of internal structures within the whipping-cream emulsions [10].

The power-law model described the steady-shear behavior of all samples, with R^2^ values ranging from 0.963 ± 0.005 to 0.994 ± 0.001 (Table 2). All mean flow behavior index values were below 1 (*n* = 0.259–0.426), quantitatively confirming the shear-thinning behavior observed in Figure 4. Within the phosphorylated-inulin series, the consistency index increased from 1.586 ± 0.175 Pa·s^n^ for PI-3 to 49.296 ± 1.113 Pa·s^n^ for PI-12, consistent with the increase in apparent viscosity at higher phosphorylated-inulin addition levels. NC showed the lowest consistency index, whereas CWC exhibited an intermediate value and was considered only as an external commercial reference.

Previous studies have similarly shown that native and modified polysaccharides can increase emulsion viscosity. Zhao et al. [12] also reported that incorporating hydroxypropyl methylcellulose (HPMC) into whipped cream led to a significant increase in viscosity. It is worth noting that, at the equivalent addition level of 12 g/batch, the whipping emulsion formulated with phosphorylated inulin (PI-12) exhibits higher viscosity than that prepared with native inulin (NI-12). This result is consistent with Chen et al. [14], who reported greater water-holding capacity and viscosity in phosphorylated-inulin gels than in native-inulin gels. The higher viscosity observed here may therefore be associated with phosphorylation-induced changes in molecular characteristics and hydration behavior [14,37]. NC, which contained no added inulin, exhibited the lowest viscosity. The viscosity of CWC fell within the range observed for some laboratory-prepared formulations; however, CWC was included only as a commercial reference because of its different composition.

#### 3.4.2. Strain Sweep

**Figure 5:**
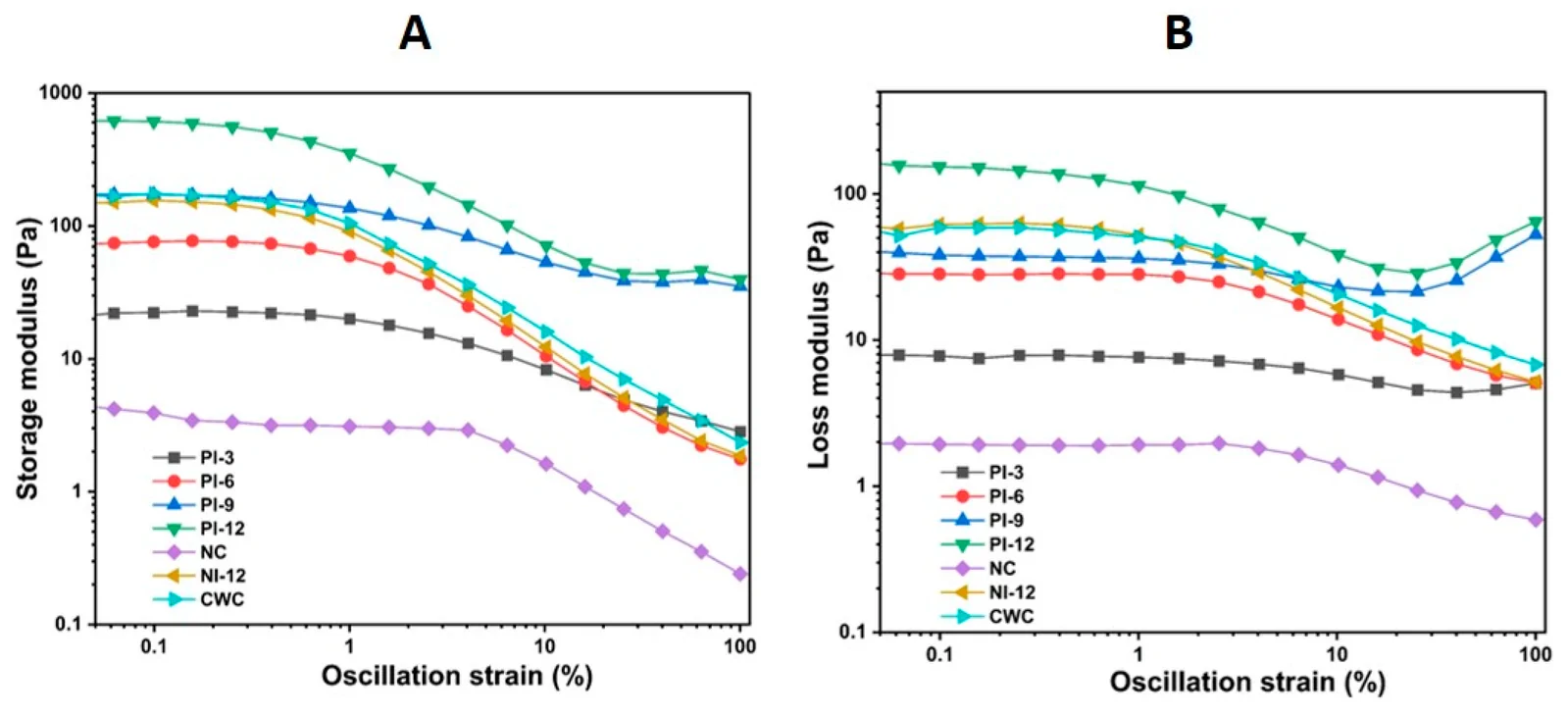
Oscillatory strain-sweep profiles of duck-fat-based whipping-cream emulsions: (**A**) storage modulus (G′) and (**B**) loss modulus (G″). Samples included the formulation without inulin (NC), formulations containing phosphorylated inulin (PI-3–PI-12), the formulation containing native inulin (NI-12), and commercial whipping cream (CWC).

Strain-sweep measurements were conducted to determine the linear viscoelastic region (LVR) of each formulation, as shown in Figure 5. Strain-sweep measurements characterize the response of an emulsion to increasing oscillatory deformation and identify the strain range within which its viscoelastic response remains linear [38]. Within the LVR, G′ remained greater than G″ for all formulations, indicating an elastic-dominant, gel-like response consistent with the findings of Cui et al. [10]. Based on the 5% decrease criterion for (G′), the estimated upper LVR limits were 0.880%, 0.580%, 0.284%, 0.169%, 0.064%, 0.312%, and 0.312% for PI-3, PI-6, PI-9, PI-12, NC, NI-12, and CWC, respectively. NC exhibited the narrowest LVR, indicating the earliest onset of nonlinear behavior. Among the phosphorylated-inulin samples, the upper LVR limit decreased from PI-3 to PI-12, showing that the higher-addition formulations entered the nonlinear region at lower strains. However, this should not be interpreted as a reduction in their small-deformation viscoelastic strength, because the magnitudes of both (G′) and (G″) increased with phosphorylated-inulin addition level.

The progressive increase in G′ and G″ from PI-3 to PI-12 indicates that increasing the phosphorylated-inulin addition level produced a more pronounced gel-like response under the tested conditions. Previous studies have similarly reported that phosphorylation can modify the hydration and rheological behavior of native inulin [14]. The higher moduli may reflect enhanced hydration and phosphate-mediated polymer–water or polymer–polymer interactions [39]. However, these molecular interactions were not measured directly and therefore represent a proposed explanation for the observed rheological behavior. NI-12 and CWC exhibited intermediate viscoelastic responses, whereas NC showed the lowest (G′) and (G″) values, indicating the weakest small-deformation structure among the tested formulations. At the equivalent addition level of 12 g/batch, NI-12 exhibited lower G′ and G″ values than PI-12, indicating a stronger viscoelastic response for the phosphorylated-inulin formulation under the tested conditions. This difference may be associated with structural changes introduced by phosphorylation.

#### 3.4.3. Frequency Sweep

**Figure 6:**
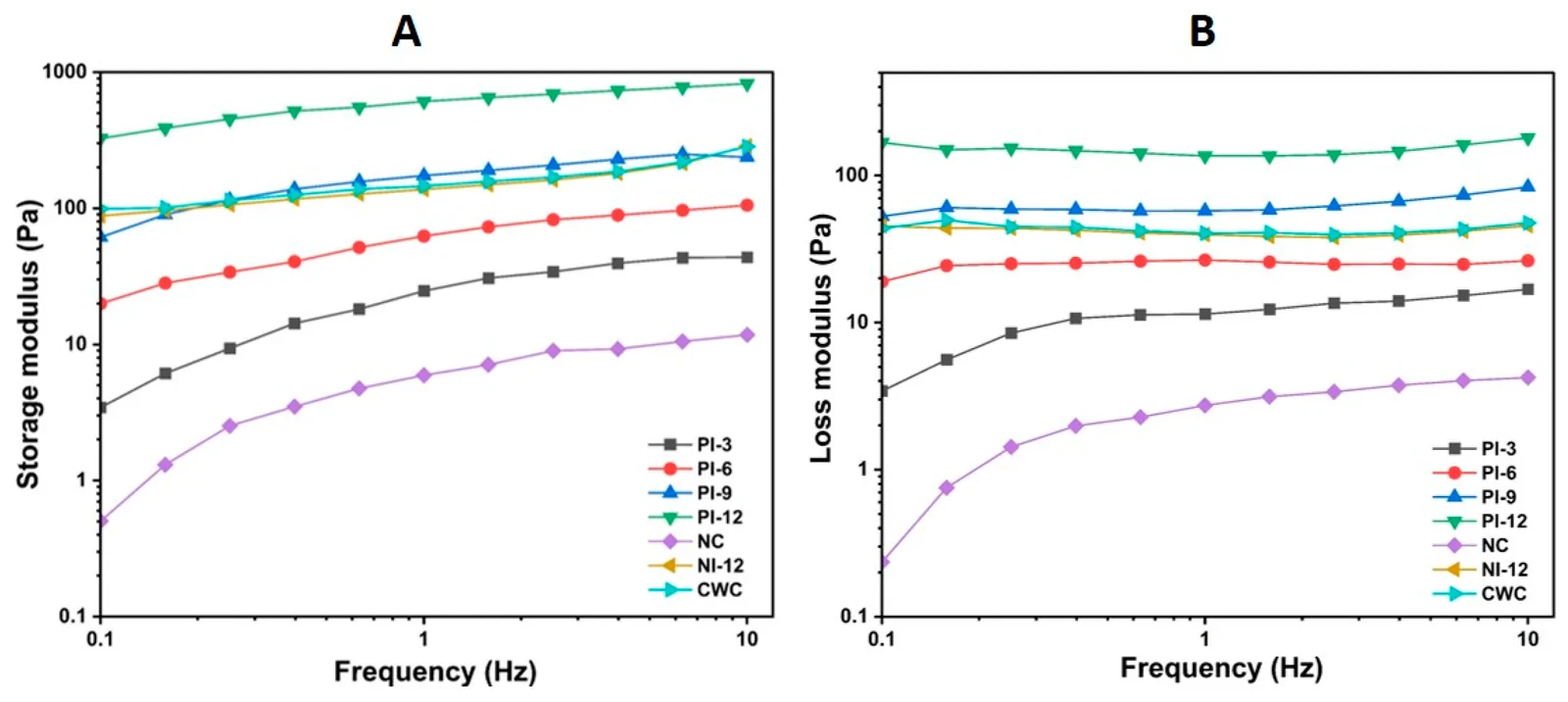
Frequency-sweep profiles of duck-fat-based whipping-cream emulsions: (**A**) storage modulus (G′) and (**B**) loss modulus (G″). Samples included the formulation without inulin (NC), formulations containing phosphorylated inulin (PI-3–PI-12), the formulation containing native inulin (NI-12), and commercial whipping cream (CWC).

Frequency sweep measurements revealed the formulation-dependent viscoelastic behavior over the tested frequency range (Figure 6). For all samples, the storage modulus G′ remained higher than the loss modulus G″, and no crossover was observed within this range, indicating a predominantly elastic response. Among the phosphorylated-inulin samples, increasing the phosphorylated inulin addition level was associated with progressively higher G′ and G″ values, generally following the order PI-12 > PI-9 > PI-6 > PI-3. PI-12 exhibited the highest moduli and comparatively lower frequency dependence, whereas NC exhibited the lowest moduli and greater frequency dependence. In contrast, NI-12 showed a broadly comparable and intermediate viscoelastic profile. Overall, higher phosphorylated-inulin addition levels were associated with a stronger viscoelastic response, while all formulations remained predominantly elastic over the measured frequency range.

The rheological differences among the PI-series samples cannot be attributed solely to phosphorylated-inulin addition, as increasing its level also increased total solids and altered the relative proportions of the other ingredients. These compositional changes may therefore have contributed to the observed increases in viscosity and viscoelastic moduli.

### 3.5. Whipping Time and Overrun

Whipping time and overrun are important factors that affect the quality and stability of whipped cream, ultimately determining its overall performance [40]. Generally, a shorter whipping time and sufficiently high overrun are desirable because they indicate efficient whipping and adequate air incorporation. However, these characteristics should be achieved without compromising foam structure, shape retention, or resistance to serum drainage. The whipping process causes fat droplets to collide and partially coalesce due to mechanical shear. Consequently, factors such as the collision rate and the degree of fat globule agglomeration may influence whipping time [41]. The whipping time increased with the phosphorylated inulin addition level (Table 3), with PI-12 exhibiting the longest whipping time (390 ± 6 s). The prolonged whipping time of PI-12 may be attributed to its higher viscosity, which increased flow resistance and limited air incorporation during whipping. In contrast, the negative-control sample (NC), which contained no added stabilizer, exhibited the shortest whipping time (105 ± 3 s). NI-12 exhibited a whipping time of 226 ± 6 s, similar to that observed for PI-9. CWC had a whipping time of 181 ± 3 s and is reported separately as an external commercial reference.

Overrun is an indicator of whipping efficacy in whipped cream, measuring the extent of air incorporation and the resulting increase in volume [8,11]. Increasing the phosphorylated-inulin addition level was associated with a reduction in overrun, from 287 ± 8% in PI-3 to 192 ± 5% in PI-12. (Table 3). The lower overrun observed for PI-9 and PI-12 may therefore be associated with their higher viscosity and viscoelasticity, which could have restricted air incorporation during whipping [42]. PI-6 exhibited an overrun of 253 ± 8%, whereas NI-12 exhibited an overrun of 219 ± 5%. CWC showed an overrun of 210 ± 5% and is reported only as a commercial reference. The NC sample exhibited the highest overrun of 331 ± 8%, possibly due to the absence of stabilizing agents. Similar reductions in overrun with increasing hydrocolloid levels have been reported in whipped cream formulations containing locust bean gum/λ-carrageenan [43], yellow mustard-fenugreek gum [11], and microcrystalline cellulose and xanthan gum [44]. The addition of hydrocolloids can increase the rheological strength of the continuous phase, thereby limiting air incorporation, prolonging whipping time, and reducing overrun.

The observed changes in whipping time and overrun should not be attributed solely to phosphorylated-inulin addition. Adding phosphorylated inulin without a corresponding reduction in water increased total solids and altered the relative ingredient composition, which may also have influenced whipping behavior.

### 3.6. Serum Loss

Reduced serum loss is indicative of enhanced foam stability. After 3 h at 22 °C, measurable serum loss was observed for NC (10.06 ± 1.03%), CWC (8.77 ± 0.95%), and PI-3 (3.23 ± 0.31%), whereas no measurable serum loss was detected for PI-6, PI-9, PI-12, and NI-12. (Table 3). The relatively low viscosities of NC and PI-3 may have contributed to their greater serum loss. Lower viscosities of these samples may facilitate air incorporation and provide less resistance to liquid drainage, thereby contributing to instability [45]. Under the specified test conditions, both PI-12 and NI-12, formulated with 12 g/batch of phosphorylated and natural inulin, respectively, showed no measurable serum loss following incubation at 22 °C for 3 h. Within the phosphorylated-inulin series, PI-6 was the lowest tested addition level at which no measurable serum loss was detected, indicating improved short-term resistance to serum drainage. This stability is likely due to the high water-holding capacity of phosphorylated inulin. Similar findings have been reported by Chen et al. [14], who prepared gels with native and phosphorylated inulin and observed that, at the same concentration, phosphorylated inulin exhibited a higher water-holding capacity compared to native inulin. Moreover, the higher viscosities observed for PI-6, PI-9, PI-12, and NI-12 may have promoted the formation of a more structured continuous phase, thereby enhancing water immobilization and limiting serum drainage. This study concludes that adding phosphorylated inulin may stabilize the continuous phase, which is in agreement with other hydrocolloid application studies [9,11,43]. By increasing aqueous-phase viscosity, these polysaccharides may promote the development of a structured continuous-phase network that limits liquid drainage and bubble mobility, thereby contributing to enhanced foam stability [46].

### 3.7. Microstructure of Whipped Cream

**Figure 7:**
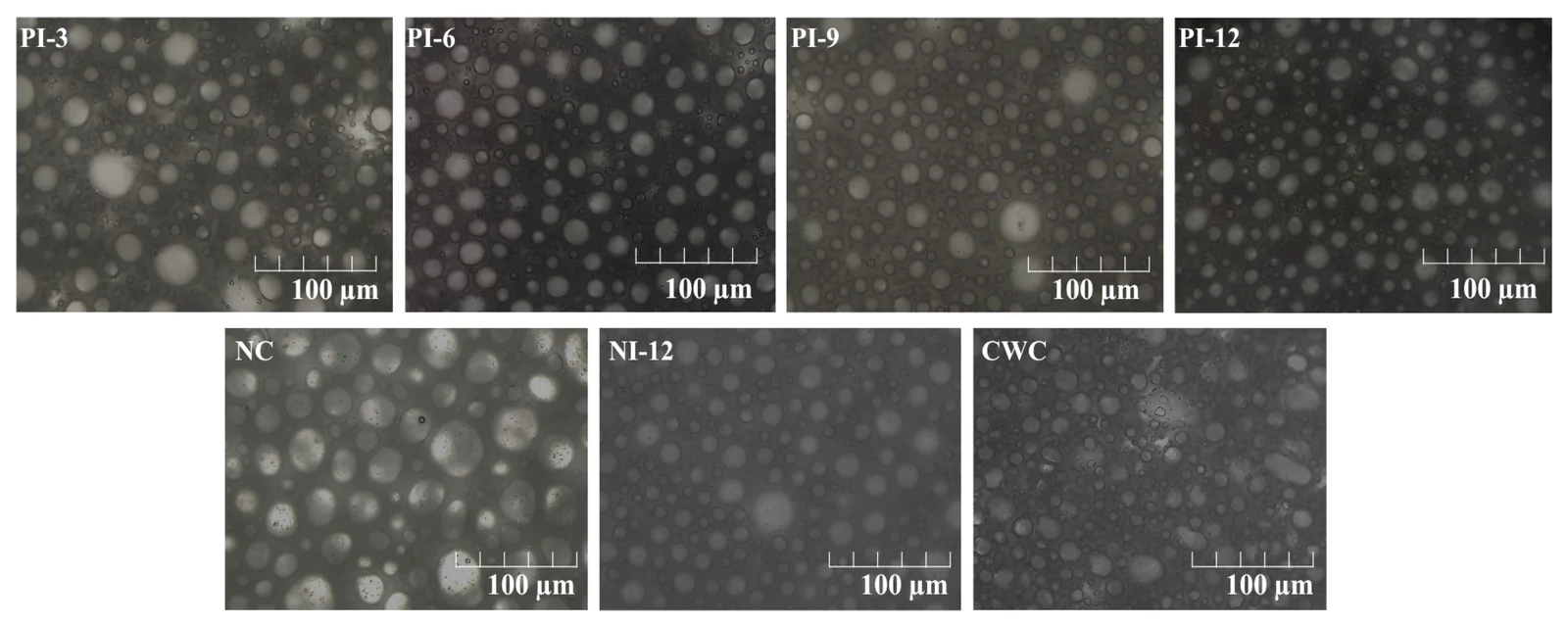
Representative optical micrographs of duck-fat-based whipped creams showing air-bubble morphology and spatial distribution. Samples included the formulation without inulin (NC), formulations containing phosphorylated inulin (PI-3–PI-12), the formulation containing native inulin (NI-12), and commercial whipped cream (CWC). Images were obtained at 40× magnification. Scale bar = 100 μm.

The stability of whipped cream is largely governed by the size and spatial distribution of air bubbles [47]. Optical microstructural analysis showed a more uniform spatial distribution of air bubbles with increasing addition levels of phosphorylated inulin. (Figure 7). At the whipping endpoint, the NC sample contained larger air bubbles that appeared less densely arranged and more unevenly dispersed. Similarly, the air bubbles in the whipped cream sample PI-3 were uneven and larger. The presence of large and unevenly distributed bubbles may increase susceptibility to coalescence and foam destabilization, consistent with the greater serum loss observed for NC and PI-3. However, air bubbles became smaller and more densely arranged as phosphorylated inulin addition level increased. Higher phosphorylated-inulin content was associated with longer whipping times, which may have contributed to the formation of smaller air bubbles. Increasing the phosphorylated-inulin content may strengthen the internal network and support air-bubble stabilization despite the observed reduction in overrun. In addition to phosphorylated-inulin addition, the concurrent increase in total solids and changes in relative ingredient proportions may have contributed to the observed differences in bubble size and foam structure. The air bubbles appeared smaller and more uniformly distributed in PI-6, PI-9, and PI-12 than in formulations with lower phosphorylated-inulin addition levels. Smaller and more uniformly distributed air bubbles are generally associated with improved foam stability [48]. It is noteworthy that in the absence of phosphorylated inulin or at a low concentration (PI-3), the whipping process was characterized by a rapid foaming rate and short whipping time. However, the bubbles generated under these conditions were undesirably large, unevenly distributed, and structurally unstable.

### 3.8. Shaping Ability and Cross Section View

**Figure 8:**
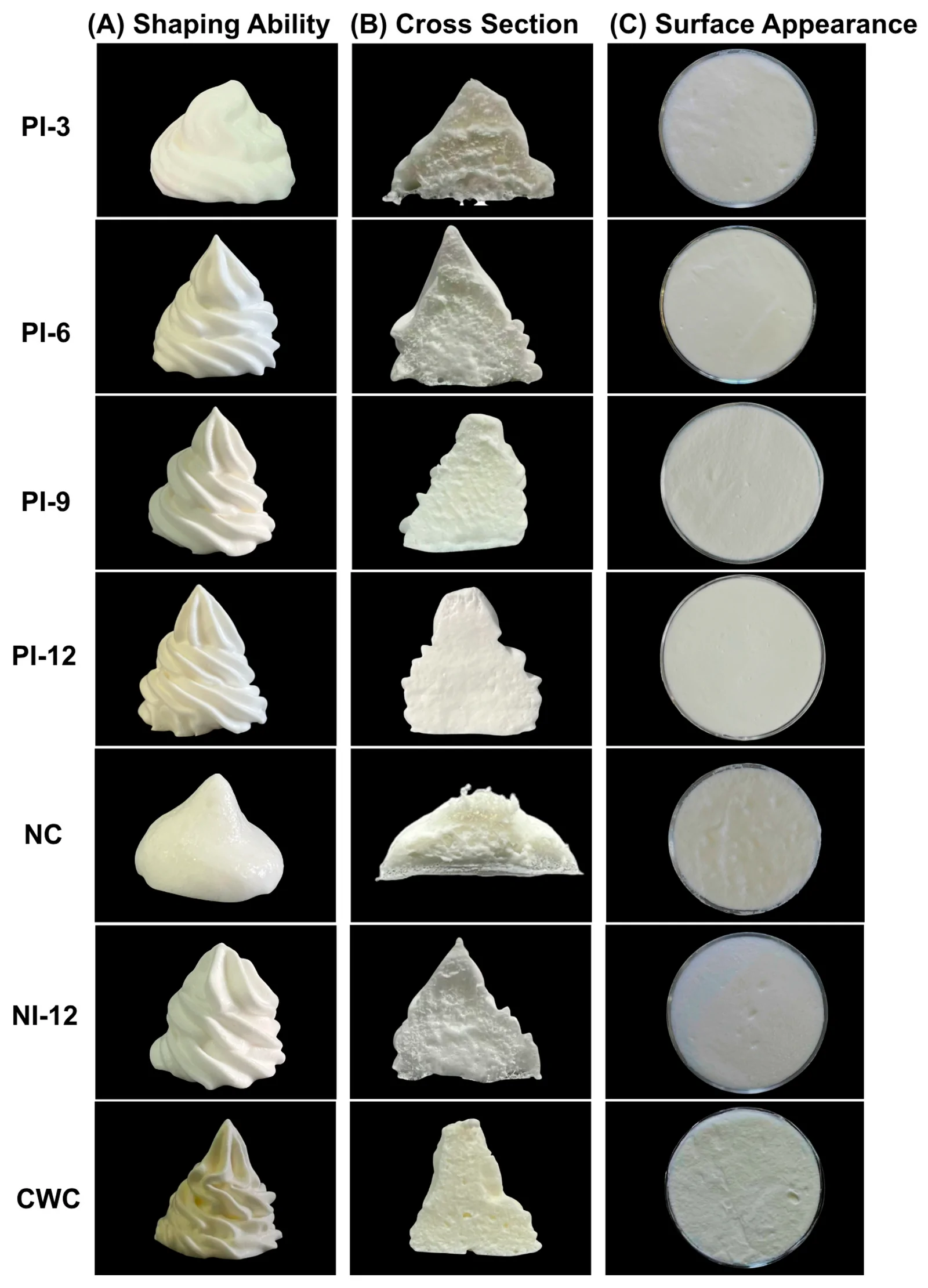
Representative photographs showing (**A**) shaping ability, (**B**) cross-sectional appearance, and (**C**) surface appearance of the whipped-cream samples. Samples included the duck-fat-based formulation without inulin (NC), formulations containing phosphorylated inulin (PI-3–PI-12), the formulation containing native inulin (NI-12), and commercial whipped cream (CWC).

The stability of whipped cream may also be assessed by its shaping ability, which serves as a primary quality indicator for whipped cream systems [10,45]. As shown in Figure 8, the shaping ability of whipped cream improved with increasing the addition level of phosphorylated inulin. NC and PI-3 exhibited the least-defined shapes. This observation was consistent with their comparatively high serum loss, coarse foam appearance, and larger visible air bubbles. The relatively low viscosity and high overrun of these samples may have contributed to their limited shape definition [45]. Compared with PI-3, samples PI-6, PI-9, and PI-12 appeared to have more clearly defined edges and smoother surfaces. Qualitative visual assessment further suggested that PI-12 and NI-12, each containing 12 g/batch of the respective inulin type, had comparable shaping characteristics. The higher viscosity observed at increased phosphorylated-inulin addition levels may have contributed to improved shape definition and a smoother decorative appearance [46].

Smoother and more uniform cross-sectional surfaces generally reflect better structural stability in whipped cream [41,49]. The cross-sectional morphologies displayed in Figure 8 reveal a clear trend. Samples with low or no phosphorylated inulin exhibited a coarse surface texture. In contrast, the cross-sectional surfaces appeared smoother and more refined as the phosphorylated-inulin addition level increased. These qualitative observations suggest that increasing the phosphorylated-inulin concentration influenced the cross-sectional appearance of the whipped cream under the tested conditions.

### 3.9. Sensory Evaluation

Four key sensory attributes of whipped cream, such as texture, overall flavor quality, sweetness, and smoothness were evaluated, and the results are presented in Table 4. These results are presented as preliminary sensory observations, and the discussion focuses on descriptive patterns in the mean scores. The PI-3 and negative control (NC) samples received the lowest mean texture scores due to their weak structure and poor shaping characteristics. Within the phosphorylated-inulin series, PI-6, PI-9, and PI-12 received higher mean texture scores than PI-3. This improvement was broadly consistent with the rheological results, in which the higher-addition formulations exhibited greater viscosity and storage modulus (G′). Furthermore, microscopic observations revealed a more uniform and compact foam structure at higher phosphorylated inulin addition levels, which contributed to the improved firmness and texture perceived by the sensory panel.

In terms of overall flavor quality, the PI-3 and NC samples received the lowest mean scores. The mean overall flavor-quality scores showed an increasing pattern across the phosphorylated-inulin series, with PI-6, PI-9, and PI-12 receiving higher mean scores than PI-3. CWC received the highest mean sweetness score. No sucrose was added to the laboratory-prepared formulations, which may partly explain their lower sweetness scores. However, CWC differed in sugar content and several other compositional characteristics and was therefore treated only as a commercial reference rather than as a directly comparable formulation. Mean smoothness scores were also higher for the higher-addition PI formulations. This descriptive pattern was broadly consistent with their more homogeneous foam appearance and greater viscoelastic response. Overall, preliminary sensory observations were consistent with the rheological and microstructural findings. However, they should not be interpreted as confirmatory evidence of significant or population-level differences in consumer acceptance because assessor-level variability could not be modelled.

Because residual free phosphate and urea were not analytically quantified, the sensory results should be regarded as preliminary observations and should not be interpreted as confirmation of the safety or broader food applicability of phosphorylated inulin.

## 4. Conclusions

This study demonstrated the potential of phosphorylated inulin as a structuring and stabilizing ingredient in duck-fat-based whipped cream. Successful phosphorylation was supported by a phosphorus content of 0.38%, a degree of substitution of 0.020, and phosphate-associated FTIR bands at approximately 1220 and 992 cm^−1^. Increasing the phosphorylated-inulin addition level was associated with higher viscosity and viscoelastic moduli, smaller apparent emulsion droplets at the higher addition levels, and improved short-term resistance to serum drainage. PI-6 (6 g/batch) was the lowest tested phosphorylated-inulin addition level at which no measurable serum loss was detected after 3 h at 22 °C, whereas PI-9 and PI-12 also showed no measurable serum loss. Higher addition levels further enhanced several rheological, microstructural, shaping, and sensory characteristics but were accompanied by longer whipping times and lower overrun, indicating a trade-off between continuous-phase structuring and whipping performance. At the equivalent 12 g/batch addition level, PI-12 exhibited a stronger viscoelastic response and smaller average particle size than NI-12 under the tested conditions. Overall, phosphorylated inulin showed promise for improving the technological performance of duck-fat-based whipped cream, particularly its short-term physical stability. Further studies should evaluate refrigerated storage stability, oxidative stability, molecular-weight changes, residual phosphorylation reagents, consumer acceptance, and scale-up performance to establish its broader applicability.

## Figures and Tables

**Table 1:** Part A: Actual ingredient quantities used to prepare each experimental formulation. Part B: Composition normalized to 100 g of final formulation.

**Part A**						
**Ingredient (g/Batch)**	**NC**	**PI-3**	**PI-6**	**PI-9**	**PI-12**	**NI-12**
Deionized water	56.17	56.17	56.17	56.17	56.17	56.17
Fractionated duck fat	29.50	29.50	29.50	29.50	29.50	29.50
Sodium caseinate	0.50	0.50	0.50	0.50	0.50	0.50
Polyglycerol fatty acid ester	0.10	0.10	0.10	0.10	0.10	0.10
Mono- and diglycerides	0.50	0.50	0.50	0.50	0.50	0.50
Phosphorylated inulin	0.00	3.00	6.00	9.00	12.00	0.00
Natural inulin	0.00	0.00	0.00	0.00	0.00	12.00
Final batch mass	86.77	89.77	92.77	95.77	98.77	98.77
**Part B**						
**Ingredient (g/100 g)**	**NC**	**PI-3**	**PI-6**	**PI-9**	**PI-12**	**NI-12**
Deionized water	64.73	62.57	60.55	58.65	56.87	56.87
Fractionated duck fat	34.00	32.86	31.80	30.80	29.87	29.87
Sodium caseinate	0.58	0.56	0.54	0.52	0.51	0.51
Polyglycerol fatty acid ester	0.12	0.11	0.11	0.10	0.10	0.10
Mono- and diglycerides	0.58	0.56	0.54	0.52	0.51	0.51
Phosphorylated inulin	0.00	3.34	6.47	9.40	12.15	0.00
Natural inulin	0.00	0.00	0.00	0.00	0.00	12.15
Total	100.00	100.00	100.00	100.00	100.00	100.00

**Note (A):** NC, negative control without inulin; PI-3 to PI-12, formulations prepared by adding 3–12 g phosphorylated inulin per batch; NI-12, formulation prepared by adding 12 g native inulin per batch. All other ingredient quantities were held constant. **Note (B):** Values were calculated from the actual ingredient quantities reported in Part A and are expressed as g/100 g of final formulation. During experimental preparation, the absolute masses of water, duck fat, sodium caseinate, polyglycerol fatty acid ester, and mono- and diglycerides were held constant. Because inulin was added without replacing water, the final batch mass increased with inulin addition; consequently, normalization to 100 g resulted in differences in the relative proportions of the fixed base ingredients. Values may not sum exactly to 100.00 due to rounding.

**Table 2:** Power-law parameters describing the steady-shear rheological behavior of the formulations.

Sample	Consistency Index, K (Pa·s^n^)	Flow Behavior Index, *n*	R^2^
PI-3	1.586 ± 0.175	0.259 ± 0.048	0.963 ± 0.005
PI-6	5.393 ± 0.593	0.426 ± 0.026	0.986 ± 0.005
PI-9	16.005 ± 0.132	0.261 ± 0.003	0.994 ± 0.001
PI-12	49.296 ± 1.113	0.267 ± 0.008	0.984 ± 0.008
NC	1.161 ± 0.109	0.329 ± 0.018	0.980 ± 0.012
NI-12	5.033 ± 1.000	0.399 ± 0.056	0.967 ± 0.028
CWC	4.399 ± 0.688	0.376 ± 0.028	0.975 ± 0.012

Note: The data were fitted to the power-law model $\tau =K{\dot{\gamma }}^{n}$ over the shear-rate range of 0.1–100 s^−1^, where K is the consistency index and n is the flow behavior index. R^2^ represents the coefficient of determination for the log–log regression. Values are presented as mean ± standard deviation of three separately fitted flow curves (*n* = 3).

**Table 3:** Whipping properties of duck-fat-based whipped creams. Samples included the formulation without inulin (NC), formulations containing phosphorylated inulin (PI-3–PI-12), the formulation containing native inulin (NI-12), and commercial whipping cream (CWC). Serum loss was measured after 3 h of incubation at 22 °C and is expressed as a percentage (%).

Samples	Whipping Time (s)	Overrun (%)	Serum Loss (%)
PI-3	124 ± 5 ^e^	287 ± 8 ^b^	3.23 ± 0.31
PI-6	170 ± 10 ^d^	253 ± 8 ^c^	ND
PI-9	226 ± 4 ^b^	212 ± 7 ^d^	ND
PI-12	390 ± 6 ^a^	192 ± 5 ^e^	ND
NC	105 ± 3 ^f^	331 ± 8 ^a^	10.06 ± 1.03
NI-12	226 ± 6 ^b^	219 ± 5 ^d^	ND
CWC	181 ± 3 ^c^	210 ± 5 ^d^	8.77 ± 0.95

Values are expressed as mean ± standard deviation. Different superscript letters within the whipping time and overrun columns indicate significant differences among samples (*p* < 0.05). ND indicates that no measurable serum loss was detected under the experimental conditions.

**Table 4:** Sensory evaluation scores of duck-fat-based whipped creams. Samples included the formulation without inulin (NC), formulations containing phosphorylated inulin (PI-3–PI-12), the formulation containing native inulin (NI-12), and commercial whipped cream (CWC). Texture, overall flavor quality, sweetness, and smoothness were evaluated using a 5-point acceptance scale (1 = poor, 5 = excellent).

Samples	Texture	Overall Flavor Quality	Sweetness	Smoothness
PI-3	2.22 ± 0.12 ^d^	2.23 ± 0.10 ^e^	1.65 ± 0.09 ^e^	2.52 ± 0.07 ^e^
PI-6	4.35 ± 0.10 ^c^	3.45 ± 0.12 ^d^	2.58 ± 0.06 ^d^	3.77 ± 0.05 ^d^
PI-9	4.56 ± 0.10 ^b^	3.80 ± 0.09 ^c^	3.05 ± 0.09 ^c^	4.19 ± 0.06 ^c^
PI-12	4.74 ± 0.08 ^a^	4.15 ± 0.09 ^b^	3.32 ± 0.10 ^b^	4.54 ± 0.10 ^b^
NC	1.65 ± 0.12 ^e^	1.31 ± 0.08 ^f^	1.30 ± 0.11 ^f^	1.17 ± 0.10 ^f^
NI-12	4.52 ± 0.09 ^b^	4.22 ± 0.07 ^b^	3.18 ± 0.07 ^c^	4.31 ± 0.06 ^c^
CWC	4.81 ± 0.07 ^a^	4.86 ± 0.05 ^a^	4.77 ± 0.05 ^a^	4.74 ± 0.06 ^a^

Values are expressed as the mean ± standard deviation of three session-level means. Different superscript letters within the same column indicate differences according to the exploratory analysis of the session-level means (*p* < 0.05). Because assessor-level variability could not be modelled, these comparisons should be interpreted cautiously and do not establish population-level differences in consumer acceptance.

## Data Availability

The original contributions presented in this study are included in the article; further inquiries can be directed to the corresponding author.
